# Overview of Mitochondrial E3 Ubiquitin Ligase MITOL/MARCH5 from Molecular Mechanisms to Diseases

**DOI:** 10.3390/ijms21113781

**Published:** 2020-05-27

**Authors:** Isshin Shiiba, Keisuke Takeda, Shun Nagashima, Shigeru Yanagi

**Affiliations:** 1 Laboratory of Molecular Biochemistry, School of Life Sciences, Tokyo University of Pharmacy and Life Sciences, Hachioji, Tokyo 192-0392, Japan; isshin.shiiba@gakushuin.ac.jp (I.S.); stakeda@toyaku.ac.jp (K.T.); nagashi@toyaku.ac.jp (S.N.); 2 Laboratory of Molecular Biochemistry, Department of Life Science, Faculty of Science, Gakushuin University, 1-5-1 Mejiro, Toshima-ku, Tokyo 171-8588, Japan

**Keywords:** mitochondria, E3 ubiquitin ligase, MITOL/MARCH5

## Abstract

The molecular pathology of diseases seen from the mitochondrial axis has become more complex with the progression of research. A variety of factors, including the failure of mitochondrial dynamics and quality control, have made it extremely difficult to narrow down drug discovery targets. We have identified MITOL (mitochondrial ubiquitin ligase: also known as MARCH5) localized on the mitochondrial outer membrane and previously reported that it is an important regulator of mitochondrial dynamics and mitochondrial quality control. In this review, we describe the pathological aspects of MITOL revealed through functional analysis and its potential as a drug discovery target.

## 1. Introduction

There are many functional proteins on the mitochondrial outer membrane. Not only mitochondrial quality control, but also various types of signal transduction including in the innate immune response, is performed on mitochondria as a scaffold. Mitochondria are dynamic organelles that form diverse networks through morphological changes via their fusion and fission, their intracellular movement along microtubules, and their interaction with other organelles. Since mitochondrial function is tightly regulated by mitochondrial dynamics, its molecular mechanisms and their association with disease have attracted substantial attention. In the ubiquitylation system, E3 ubiquitin ligases play a key role in determining substrate specificity and catalyzing the transfer of ubiquitin from E2 enzymes to the substrate. Growing evidence has shown that E3 ubiquitin ligases are involved in the regulation of mitochondrial functions. HECT domain type E3 ligases have one more transthiolation reaction to transfer the ubiquitin onto the E3, whereas the much more common RING finger domain type ligases transfer ubiquitin directly from E2 to the substrate. MITOL (mitochondrial ubiquitin ligase: also known as MARCH5) is a mitochondrial membrane-associated RING finger E3 ubiquitin ligase and was initially identified as a regulator of mitochondrial dynamics, which involves two different aspects: the regulation of protein activation by K63 ubiquitin chain attachment and the regulation of proteasome-dependent degradation by K48 ubiquitin chain attachment via ubiquitin signaling on the outer mitochondrial membrane. A number of MITOL substrates have been identified so far and their association with disease has been suggested. In this review, we present the latest findings on MITOL as a potential drug target and its relevance to disease and aging.

## 2. Mitochondria and Intracellular Quality Control by MITOL

### 2.1. Neurodegenerative Diseases

Various quality control systems enable the production of high-quality proteins in the cell. The endoplasmic reticulum is equipped with a protein quality control mechanism termed endoplasmic reticulum-associated degradation (ERAD), in which the ubiquitin–proteasome system (UPS) plays a central role [1]. The UPS selectively and rapidly degrades and removes abnormal proteins by adding ubiquitin chains [2]. Recently, a new quality control mechanism called mitochondrial protein translocation-associated degradation (mitoTAD) has been discovered in yeast [3]. It would be interesting to determine whether such a sophisticated quality control mechanism is preserved in mammals. The accumulation of dysfunctional proteins has been observed in many neurodegenerative diseases, and it has become clear that it is deeply involved in their pathogenesis. The increase in dysfunctional proteins due to mutations in the causative gene and the decrease in proteasome activity with aging are thought to be the main pathogenic factors. The accumulation of dysfunctional proteins in cells leads to a decrease in proteasome activity, suggesting the presence of a downward spiral that further promotes the accumulation of aggregated proteins [4]. It has been reported that such highly aggregated dysfunctional proteins can also accumulate in mitochondria and impair their function [5]. As an example, mutant superoxide dismutase-1 (mSOD1), one of the gene products responsible for amyotrophic lateral sclerosis (ALS), aggregates due to its conformational changes, accumulates in mitochondria, and inhibits mitochondrial protein transport, thereby impairing mitochondrial function. In addition, in spinal cerebellar degeneration, such as polyglutamine disease, the abnormal elongation of the polyglutamine chain resulting in conformational changes leading to accumulation of the protein PolyQ in the nucleus and mitochondria, and reduces mitochondrial function, which is thought to be one of the mechanisms of neuropathy. We reported that MITOL specifically ubiquitinates and promotes the proteasomal degradation of the misfolded proteins mSOD1 and PolyQ as substrates [6,7]. MITOL has a disordered domain in its C-terminal region, which may recognize denatured proteins. This suggests that MITOL is involved in mitochondrial quality control by eliminating dysfunctional proteins from the mitochondria. However, it is controversial whether MITOL recognizes target proteins in the process of aggregation or after their aggregation.

### 2.2. Innate Immune Response

**Figure 1:**
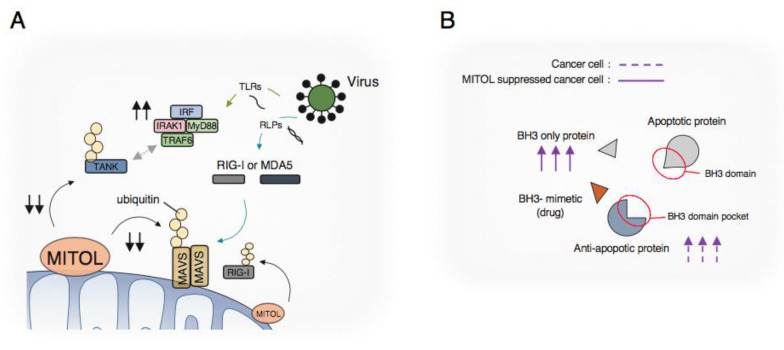
(**A**) MITOL regulates both RIG-I-like receptor (RLR) pathway and Toll-like receptor (TLR) pathway; (**B**) Relativity between MITOL and cell death signaling in cancer cells.

In mammals, the innate immune system against RNA viruses is regulated by two different signaling pathways: the TLR (Toll-like receptor) pathway and the RLR (RIG-I-like receptor) pathway. The membrane-associated RING-CH (MARCH) family of E3 ubiquitin ligases is thought to perform immunoregulatory functions by controlling the localization and abundance of immune receptors [8]. In the RLR pathway, RIG-I/MDA-5 interacts with mitochondrial antiviral signaling (MAVS), also known as VISA, on mitochondria as an adaptor and transmits signals downstream. MITOL, the only member of the MARCH family localized in mitochondria, prevents excessive immune responses by ubiquitinating phosphorylated MAVs and activated RIG-I for degradation [9,10,11]. In addition, MITOL acts as a positive modulator in the TLR pathway, particularly through the ubiquitination of TANK, a negative regulator of TLR7 signaling [12]. Furthermore, MITOL preserves mitochondrial function by ubiquitinating HBx, which is localized in mitochondria for degradation in response to hepatitis B virus [13]. These findings suggest that MITOL could regulate immunosignaling on mitochondria (Figure 1A).

### 2.3. Cell Death in Cancer Cells

Mitochondria store apoptotic factors such as cytochrome c, Apoptosis-inducing factor (AIF), and caspase-9 in the intermembrane space and release them from the mitochondria to the cytoplasm in response to extracellular death signals, which initiates a cascade of apoptosis. The mitochondrial membrane permeability of cytochrome c is regulated by the B-cell leukemia gene-2 (Bcl-2) family, with pro-apoptotic proteins (Bax and Bak with BH3 domain) and anti-apoptotic proteins (Bcl-2, Bcl-xL, and Mcl-1 with BH3 binding site). There are also BH3-only proteins (Bim, Bid, Bad, Noxa, and Puma) that have only a BH3 domain among the Bcl-2 family members. These BH3-only proteins induce apoptosis by binding to the anti-apoptotic member bcl-2 and counteracting its apoptotic inhibitory effect. Mechanistically, NOXA/Mcl-1 complex was basically ubiquitinated by MITOL for degradation, cooperating with MTCH2 in a steady state [14]. Because many cancer cells escape cell death by activating the anti-apoptotic pathway such as via the upregulation of Mcl-1, BH3 mimetics are used for cancer treatment to inhibit anti-apoptotic activity and promote cell death [15].

At first glance, the activation of MITOL appears to be effective for cancer treatment. However, the quantity of NOXA is important for the promotion of cell death in cancer cells treated with ABT737 (BH3 mimetic), suggesting that the suppression of MITOL is effective to promote cancer cell death, resulting in the upregulation of NOXA [16,17]. As it was also shown that the overexpression of MITOL in breast cancer cells promotes tumor growth and metastasis [18], the suppression of MITOL might be effective for cancer treatment (Figure 1B).

### 2.4. Mitophagy

Mitophagy, a mitochondrion-specific type of autophagy, is responsible for removing damaged mitochondria and protecting cells from injury. PTEN-induced putative protein kinase 1 (PINK1) and Parkin are key players in the regulation of ubiquitin-dependent mitophagy [19]. In healthy mitochondria, PINK1 is transported into the inner mitochondrial membrane (IMM), where it is processed and cleaved by several proteases [20,21]. The truncated form of PINK1 is degraded by the ubiquitin–proteasome system [20,21]. Following the loss of the mitochondrial membrane potential, mitochondrial import is prevented, facilitating PINK1 stabilization on the outer mitochondrial membrane (OMM) [20,21,22]. PINK1 is activated by auto-phosphorylation leading to the translocation of Parkin to the mitochondrial surface. In this process, MITOL plays a role in the initial step in Parkin recruitment to mitochondria, resulting in ubiquitination of its substrate [23]. Although the physiological significance of this is still unclear, it has also been shown that MITOL is transferred to other organelles during mitophagy [24]. We have actually observed translocation to other organelles as well. Several mitochondrial proteins, including Nix/BNIP3L, BNIP3, FUNDC1, Optineurin, NDP52, and Bcl2-L-13, have been reported as mitophagy receptors in mammalian cells [19]. In particular, FUNDC1 is thought to be involved in mitophagy through its interaction with LC3 (yeast Atg8 ortholog) and ULK1 (yeast Atg1 ortholog) through hypoxic stress and inhibitory treatment of oxidative phosphorylation [25]. MITOL has been shown to regulate mitophagy by ubiquitinating FUNDC1 for degradation during hypoxic stress [26], suggesting that MITOL also acts as a regulator of such dynamic mitochondrial quality control. These observations suggest that MITOL may determine mitochondrial fate in response to a variety of mitochondrial stresses.

### 2.5. Aging

**Figure 2:**
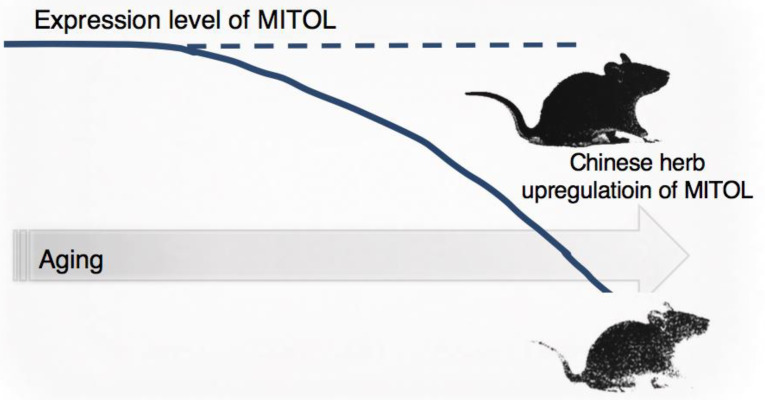
Relativity between MITOL and aging. Since MITOL gradually decrease following aging, upregulation of MITOL by a Chinese herb might be able to effect anti-aging.

Mitochondrial dysfunction has been shown to lead to oxidative stress due to the generation of excessive reactive oxygen species, triggering senescence and age-related diseases. Since cell lines with MITOL deficiency induces cellular senescence [27], we also focused on the association between reduced MITOL function and senescence and age-related diseases in vivo. In our in vivo analysis, mice lacking MITOL in a skin-specific manner showed significant signs of aging, including gray hair and hair loss. These results and MITOL downregulation during aging suggest that MITOL may block the induction of physiological aging. We hoped that drugs that upregulate MITOL expression could inhibit or delay aging, and in a collaborative study with pharmaceutical companies, we screened drugs that upregulate MITOL mRNA from a Chinese medicine library using cultured human keratinocytes. We found that extracts of *Coptis japonica* and *Phellodendron amurense* in a Chinese medicine library upregulated MITOL mRNA approximately threefold (Patent No: P2019-52145A). When berberine, common compound of *Coptis japonica* and *Phellodendron amurense*, was mixed with drinking water, the expression of mRNA and protein of MITOL increased in various organs and tissues, including skin. To investigate the anti-aging activity of berberine, mice treated with it were irradiated with ultraviolet light to induce the formation of wrinkles in the skin, and a significant inhibitory effect on this was observed compared with that in control mice. In addition, with collaboration research, we found that the mice that took berberine for more than 1 year were significantly less likely to show signs of aging, such as skin hair loss and thickening of the epidermis, than control mice. Because the anti-aging effect of berberine on mice deficient of MITOL specifically in the skin was not confirmed, anti-aging effects are induced especially via the upregulation of MITOL expression levels. These results strongly indicate that MITOL has the potential to be a target for anti-aging drugs. In the future, it is anticipated that new MITOL activators other than berberine will be identified and applied to various aging-related diseases such as neurodegenerative diseases and heart failure. Interestingly, the expression of MITOL has been observed to gradually decrease with aging in many organs such as the brain and heart of mice. Moreover, Alzbase, a database for gene dysregulation in Alzheimer’s disease (AD), suggested that the expression level of the mitol/march5 gene was significantly reduced in the brain of AD patients. If the worsening of AD is linked to the reduced MITOL expression, the compounds we have identified may exert a good therapeutic effect on AD. Therefore, we speculate that the decreased degradation of denatured proteins caused by decreased expression of MITOL may be one of the factors exacerbating the development of neurodegenerative diseases with aging (Figure 2).

## 3. Regulation of Mitochondrial Dynamics by MITOL

### 3.1. Mitochondrial Fission

**Figure 3:**
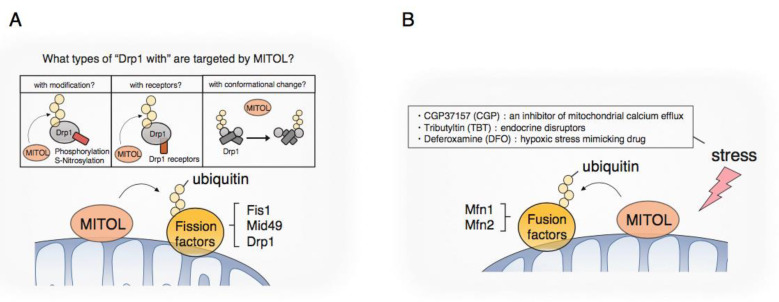
Regulation of mitochondrial fission and fusion factors by MITOL. (**A**) MITOL might have some specificity for its target; e.g., especially Drp1, such as relating to modifications, structural changes, and interactions with or without Drp1 receptors; (**B**) Alteration of substrate specificity for mitochondrial fusion factors in response to stress.

Drp1, a dynamin-related GTPase, is the central player in mitochondrial fission [28,29,30]. Drp1 basically exists in the cytosol and can be recruited to the surface of mitochondria to wrap around mitochondrial tubules. Increasing Drp1 GTP hydrolysis activity leads to conformational changes [31,32] that increase the tightness of mitochondrial constriction and the scission of mitochondrial tubules. In terms of the chemical modifications of Drp1, phosphorylation, ubiquitination, and SUMOylation by several kinases and E3 ligase, including ubiquitin and SUMO ligase, have been particularly well studied [33,34,35]. Drp1 is ubiquitylated by APC/CCdh1 E3 ubiquitin ligase complex for degradation, the regulator of the M- to G1-phase transition, for regulating mitochondrial morphology during the G1/S phase [36]. Parkin, an E3 ligase mainly localized in the cytosol in a steady state, also induces the proteasomal degradation of Drp1 [37,38], suggesting that Parkin targets Drp1 as a substrate and plays an inhibitory role in mitochondrial fission. Controversial results have been reported on functions of MITOL in regulating mitochondrial dynamics. We and others showed that MITOL interacts with Drp1 and leads to its ubiquitylation and proteasome-dependent degradation to inhibit excessive mitochondrial fission [39,40]. However, the opposite role of MITOL in mitochondrial fission, namely, its positive regulation of Drp1, was observed in another study [41]. In a later study, Karbowski’s group reported that MITOL ubiquitinates a Mid49, a Drp1 receptor, for degradation to control mitochondrial fission [42]. These findings suggest that there may be some specificity regarding regulation of MITOL for its substrates, especially Drp1, such as relating to modifications, structural changes, and interactions with or without Drp1 receptors (Figure 3A).

Recently, denitrosylase *S*-nitrosoglutathione reductase (GSNOR)-deficient mice were generated and it was reported that excessive *S*-nitrosylation resulted in the disruption of their mitochondrial dynamics and presented a typical senescence phenotype [43]. At the molecular level, it has also been reported that Drp1 can be in active form when subjected to *S*-nitrosylation by NO, leading to excessive mitochondrial division and the induction of neurotoxicity [44]. Similarly, MAP1B-LC1, a microtubule-associated factor that regulates retrograde mitochondrial transport, is also activated by *S*-nitrosylation, changing its steric structure [45], which in turn causes mitochondrial aggregation and cytotoxicity [46]. Thus, NO disrupts mitochondrial dynamics via the *S*-nitrosylation of Drp1 and MAP1B-LC1, even at the molecular level, and is thought to induce cellular damage as a result. We identified MAP1B-LC1 as a binding protein of MITOL using the yeast two-hybrid method and found that MITOL specifically recognizes and induces the degradation of *S*-nitrosylation-modified MAP1B-LC1 [47]. We also confirmed similar results in Drp1, suggesting that MITOL has selectivity for recognizing its substrates and protecting against the collapse of mitochondrial dynamics partly by degrading active Drp1 and MAP1B-LC1.

Mitochondrion–organelle contacts are involved in mitochondrial fission [48,49,50,51]. In mammals, Fis1 is a mitochondrial recruitment factor for TBC1D15, which drives lysosomal Rab7 GTP hydrolysis [52,53] and indirectly regulates mitochondrial fission at the mitochondrion–lysosome contact sites [50]. It has been reported that MITOL ubiquitinates Fis1 and promotes its degradation [39]. These findings suggest that MITOL also regulates mitochondrial fission at the mitochondrion–lysosome contact sites via Fis1. Further work examining the mechanistic role of MITOL in mitochondrion–lysosome contacts is required.

Although MITOL is clearly involved in regulating the fission machinery, we need to comprehensively consider the involvement of multiple signaling platforms explained by the diverse roles in cellular biology, variations in tissue-specific expression, and the activity of fission members for the regulation of fission by MITOL.

### 3.2. Mitochondrial Fusion

Mitochondria are fused through a few steps, termed mitochondrial fusion. First, mitochondria tether together via mitofusin (MFN), a large GTPase called Fzo1 in yeast, and MFN1/2 in mammals. Then, following MFN activation and conformational changes induced by GTP hydrolysis, each OMM attaches and fuses [54,55,56]. In recent years, various E3 ligases have been shown to regulate mitochondrial fusion via the modulation of one or both MFNs in response to various physiological or stress-induced conditions. Glycoprotein 78 (Gp78), an ER membrane-anchored E3 ubiquitin ligase, interacts with both MFNs, and Gp78 overexpression induces mitochondrial fragmentation [57]. Moreover, autocrine motility factor (AMF) prevents the Gp78-induced degradation of both MFNs [58]. MGRN1, an E3 ligase located in the cytoplasm, plasma membrane, endosomes, and nucleus, was reported to promote mitochondrial fusion via the non-degradative ubiquitylation of MFN1, consistent with previous observations [59,60]. The OMM E3 ligase MAPL/Mul1 leads to the specific ubiquitylation and degradation of MFN2 to regulate mitochondrial morphology [61]. HUWE1, a cytoplasmic E3 HECT family ubiquitin ligase also termed Mule/ARF-BP1/HectH9/E3Histone/Lasu12, ubiquitinated MFN2 associated with genotoxic stress to regulate mitochondrial fusion [62]. MITOL-mediated ubiquitylation and degradation of MFN1, but not of MFN2, lead to mitochondrial fragmentation in various inducible stresses and situations. In prostate cancer cells, the induction of cell death with CGP, an inhibitor of mitochondrial calcium efflux, led to ubiquitylation and degradation of MFN1 by MITOL [63]. It was also shown that MITOL ubiquitylates and degrades MFN1 at G2/M, the notable phase of mitochondrial fragmentation before cellular division [64]. However, under hypoxic stress induced by deferoxamine (DFO), MITOL interacts with MFN2 and is responsible for the ubiquitylation and degradation of MFN2 in cells lacking HDAC6 [65]. In conclusion, although mitochondrial fission and fusion were clearly regulated by ubiquitylation, further studies are required to understand how MITOL divides substrate-specificity according to its surroundings in order to regulate mitochondrial dynamics (Figure 3B). In addition, mitochondrial dysfunction is suspected to be one of the causes of many neurodegenerative diseases such as Alzheimer’s disease and Parkinson’s disease, so investigating the involvement of MITOL in these diseases may lead to future therapies.

## 4. Relation between MITOL and Membrane Bontact Site with the Endoplasmic Reticulum

### 4.1. Membrane Contact Site with the Endoplasmic Reticulum

The mitochondrial surface also represents the signal hub where a host of metabolic systems cross-talk through inter-organelle communication. Mitochondria indeed have a unique microdomain physically and functionally connecting to other organelles such as the endoplasmic reticulum (ER). The membrane contact site (MCS) between the ER and mitochondria is maintained by some tethering or spacer proteins such as PDZD8, Fis1-BAP31, VDAC-IP3R, PTPIP51-VAPB, and MFN2 (the tethering function of Mfn2 appears to still be controversial) in mammals [66,67]. Mitochondria and the ER can exchange lipids and calcium ion through their MCS [68,69]. The proximal domain between the ER and mitochondria is also available as a membrane scaffold for signal transmission including autophagy, inflammation, and the unfolded protein response (UPR) due to the raft-like membrane structure [70]. Taking these findings together, the ER and mitochondria complement each other through inter-organelle communication such as membrane contacts.

### 4.2. MCS Formation by MITOL

MITOL can control signals contributing to various features outside mitochondria through the MCS between the ER and mitochondria. A primary finding that MITOL is enriched in the MCS between the ER and mitochondria led us to investigate MITOL with a focus on MCS.

**Figure 4:**
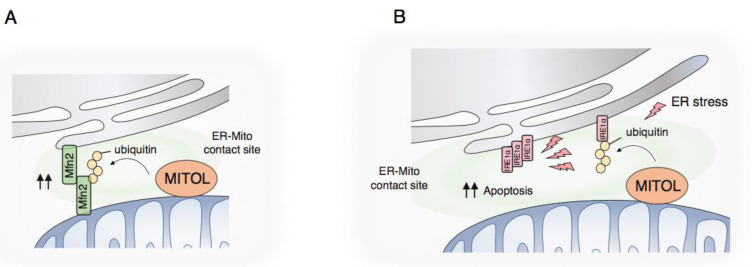
(**A**) Membrane contact site (MCS) formation by MITOL-MFN2 axis. MITOL ubiquitinates mitochondrial MFN2. The ubiquitinated MFN2 enhances its GTPase activity, triggering trans-oligomerization between mitochondrial MFN2 and ER-localized MFN2 for tethering both organelles; mitochondria and the ER; (**B**) UPR regulation by MITOL-IRE1α axis. MITOL ubiquitinates IRE1α at ER-mitochondria contact site in unstressed and low-stressed conditions regarding the ER. The ubiquitination of IRE1α does not perturbate the oligomerization itself in response to ER stress, however, leading to short-term stabilization and smaller oligomerization. In contract, the ubiquitination by MITOL is reduced under severe or chronic ER stress, resulting in continuous oligomerization of IRE1α and apoptotic switching of IRE1α signaling.

In basal and physiological conditions, MITOL interacts with and ubiquitinates the mitochondrial GTPase MFN2 [71]. MFN2 acts as a tethering factor between the ER and mitochondria, as well as a factor for inter-mitochondrial fusion via its GTPase activity [72]. The MFN2 ubiquitination by MITOL contributes to its function related to ER–mitochondrion contacts through the GTPase activation of MFN2 (Figure 4A). Thus, MFN2 mutated at K192, the lysine specific for MITOL-mediated ubiquitination, leads to the inability to connect the membrane between the ER and mitochondria, resulting in a failure of calcium ion transfer from the ER to mitochondria in the cell. The disruption of MCS between the ER and mitochondria is not fatal for the cell but affects setting the threshold for the complementation of mitochondrial or ER defects triggered by pathological conditions. Therefore, abnormal formation of the MCS might initiate or aggravate progressive, not early-developmental, diseases in humans. Actually, mutations in the MFN2 gene lead to its catalytic inactivation and subsequently trigger Charcot-Marie-Tooth disease type 2A (CMT2A) in the peripheral nervous system [73]. Similarly, a primary mutation in Sig1R or SOD1, a cause of inherited juvenile ALS, was shown to result in the disruption of MCS between the ER and mitochondria [74].

What is the in vivo and physiological contribution of MCS between the ER and mitochondria? What is triggered by the perturbation of MCS between the ER and mitochondria in vivo remains poorly characterized. There are also serious concerns regarding the accuracy of the methods for analyzing the MCS structure in vivo. To obtain an understanding of the morphology of MCS between the ER and mitochondria in vivo, electron microscopy is mostly adopted (sometimes potentially being the only method available in vivo). However, both organelles, mitochondria and the ER, exhibit complex and diverse morphology. A single image obtained from an electron microscope was limited and restricts us to evaluating a whole picture of mitochondrial states. It is also difficult to judge whether the membrane structure in a single image is part of the continuous ER or an independent part. Therefore, there is an urgent need to accurately investigate the structure of MCS between continuous ER and mitochondria in the brain.

To obtain a precise understanding of the morphology of MCS between mitochondria and the ER in neurons, we recently performed three-dimensional (3D) reconstructions from serial electron microscopy images of mitochondria using serial block—face scanning electron microscopy (SBF-SEM) [75]. Interestingly, over 95% of mitochondria had at least one contact site with the continuous ER in the brain regardless of the individual morphology of mitochondria, suggesting that MCS with the ER is pivotal for almost all mitochondria, at least in neurons (in this analysis, only neurons were selected morphologically from serial images). Larger mitochondria required more MCS with continuous ER. However, each MCS with continuous ER displayed morphological differences, such as large and small types. Contacts between the ER and mitochondria with distinct sizes might involve distinct roles. We also examined the physiological contribution of the MITOL-MFN2 axis to MCS using mice with nerve-specific ablation of MITOL. The MITOL-deleted brain showed the formation of fewer and smaller MCS between continuous ER and mitochondria. However, the phenotype of MITOL-deleted neurons regarding MCS appeared to be restricted to only larger mitochondria. When taking the findings as a whole, mitochondrial defects in the brain with MITOL deletion were mild, leading to slight developmental abnormalities in the brain. Currently, we are examining the pathological contribution of disrupted mitochondrion-ER connections during disease development by performing crossing with murine models for aging-related diseases.

### 4.3. UPR Regulation by MITOL

In addition to the basic role of MITOL in unstressed conditions, recent evidence has implied that MITOL serves as a unique signaling regulator in several specific conditions. We recently identified a novel substrate, IRE1α, for MITOL at the MCS between the ER and mitochondria [76]. IRE1α is an ER membrane-integrated protein that possesses bifunctional activity as a kinase and endoribonuclease. The luminal domain of IRE1α contributes to monitoring the stress level of the ER by sensing the emergence of unfolded proteins. Meanwhile, the cytosolic domain of IRE1α, containing both catalytic domains, contributes to signal transduction from the ER to outside it in order to recover from ER damage during ER stress conditions. The ubiquitination of IRE1α by MITOL determines the persistence of IRE1α activation upon ER stress (Figure 4B). MITOL-catalyzed ubiquitination of IRE1α is accompanied by binding to the mediator BIM for a smaller state of (or less stable) IRE1α oligomerization, allowing the catalytic activation of IRE1α only for the short term. This regulation of IRE1α is pivotal with regard to termination of the UPR after the recovery of ER homeostasis. Unresolved IRE1α activation indeed triggers an alternative outcome of IRE1α signaling, namely, cell death. Importantly, severe ER stress attenuates the ubiquitination levels of IRE1α by MITOL via unclear mechanisms. Therefore, the unlimited IRE1α oligomerization and activation promote the induction of cell death during severe and irremediable ER stress. Taking these findings together, MITOL prevents the signal switching of IRE1α from cell survival to cell death via direct ubiquitination of IRE1α at the MCS between the ER and mitochondria. The ER network accumulates severe abnormalities, both functional and morphological, during irremediable ER stress. Thus, it might be reasonable to set the monitoring system, related to the signal switch of the UPR, outside the ER, such as on the mitochondrial surface. Several mitochondrial molecules therefore hold therapeutic potential in diseases initiated or aggravated by ER stress, not only mitochondrial stress.

## 5. Discussion

It has become clear that ubiquitin not only regulates proteasome-dependent proteolysis, but also partly regulates various cellular functions such as signal transduction, membrane protein transport, selective autophagy, and aging. The multiple functions of ubiquitin are derived from the structural diversity of ubiquitin modifications, eight different linking modes, chain length, branching, and a wide variety of higher-order structures resulting from combinations of post-translational modifications of ubiquitin. Globally, the success of proteasome inhibitors in the treatment of cancer has led to substantial progress in the development of ubiquitin drugs. In particular, targeted proteolysis and induction techniques using small molecules such as PROTACs and SNIPERs are attracting substantial attention as a new generation of drug discovery methods, and there is growing momentum for the formation of groups based on the fusion of ubiquitin research and chemical biology. We expect MITOL, an E3 ubiquitin ligase, to be one of the successful examples of academic drug discovery, as it is becoming a potential target for promising seeds.
